# Supplementary material for: Ca2+-Driven Selectivity of the Effect of the Cardiotonic Steroid Marinobufagenin on Rabbit Sinoatrial Node Function
Source: Cells. 2023 Jul 18;12(14):1881. doi: 10.3390/cells12141881 (PMC10378090; doi:10.3390/cells12141881)
Supplement: Supplementary file 1 [file cells-12-01881-s001.zip › cells-2410410-supplementary/Table S1.pdf]

	Control	MBG 50nM	MBG 100nM	MBG 1000nM
**Ca^{2+} transient parameters No Change**				
**Beat Interval [ms]**	540.1±80.84 (N=6)	717.28±105.82 (N=6)	594.72±66.24 (N=6)	1038.62±354.99 (N=4)
**Beat Interval SD [ms]**	133.08±28.64 (N=6)	188.56±51.02 (N=6)	116.9±21.58 (N=6)	645.94±500.08 (N=4)
**Time to peak [ms]**	144.64±22.08 (N=6)	218.55±60.95 (N=6)	171.42±25.9 (N=6)	193.88±73.88 (N=4)
**Time to 50% relaxation [ms]**	227.55±49.87 (N=6)	328.71±78.1 (N=6)	284±54.33 (N=6)	328.52±149.66 (N=4)
**Time to 90% relaxation [ms]**	340.34±71.61 (N=6)	466.1±89.45 (N=6)	423.48±79.13 (N=6)	460.32±212.07 (N=4)
**Spontaneous diastolic LCR Characteristics No Change**				
**50% spark duration [ms]**	34.69±0.55 (N=187)	42.53±1.84** (N=178)	38.57±0.86** # (N=110)	42.2±1.17** ^ (N=110)
**Normalized amplitude [N.U]**	1.74±0.12 (N=187)	2.03±0.15 (N=178)	1.97±0.22 (N=110)	2.54±0.23** (N=110)
**Amplitude difference [N.U]**	4.48±0.4 (N=187)	9.71±0.63** (N=175)	0.25±0.38** ## (N=110)	-0.29±0.48** ## (N=110)
**Spark length [um]**	5.09±0.18 (N=187)	4.71±0.15 (N=178)	4.33±0.2** (N=110)	5.67±0.21* ## ^^ (N=110)
**LCR period [ms]**	281.52±10.91 (N=186)	372.47±16.8** (N=177)	351.99±15.12** (N=104)	676.48±72.87** ## ^^ (N=101)
**Number of LCR [1/sec*um]**	31.16±6.77 (N=6)	29.66±8.41 (N=6)	18.33±5.16* # (N=6)	27.5±7.84 (N=4)
**Ca2+ signal of individual LCR (ms*μm*F/F0)**	305.82±29.04 (N=187)	368.52±29.6 (N=178)	299.06±35.69 (N=110)	669.62±86.86** ## ^^ (N=110)
