# Supplementary material for: Ca2+-Driven Selectivity of the Effect of the Cardiotonic Steroid Marinobufagenin on Rabbit Sinoatrial Node Function
Source: Cells. 2023 Jul 18;12(14):1881. doi: 10.3390/cells12141881 (PMC10378090; doi:10.3390/cells12141881)
Supplement: Supplementary file 1 [file cells-12-01881-s001.zip › cells-2410410-supplementary/Table S2.pdf]

| | Control | MBG 50nM | MBG 100nM | MBG 1000nM |
|---|---|---|---|---|
| **$Ca^{2+}$ transient parameters Decrease** | | | | |
| **Beat Interval [ms]** | 431.46±25.79 (N=10) | 537.74±63.58 (N=10) | 677.64±89* ## (N=10) | 923.64±118.97** ## ^ (N=10) |
| **Beat Interval SD [ms]** | 71.88±12.79 (N=10) | 93.62±23.14 (N=10) | 138.51±27.33** # (N=10) | 274.1±71.13* # (N=10) |
| **Time to peak [ms]** | 135.03±10.09 (N=10) | 133.57±11.52 (N=10) | 158.75±19.61 (N=10) | 169.63±19.42 (N=10) |
| **Time to 50% relaxation [ms]** | 202.7±10.44 (N=10) | 208.03±12.82 (N=10) | 265.22±34.13 (N=10) | 253.45±50.48 (N=10) |
| **Time to 90% relaxation [ms]** | 313.8±19.39 (N=10) | 361.06±29.72 (N=10) | 451.55±70.21 (N=10) | 466.56±92.02 (N=10) |
| **Spontaneous diastolic LCR Characteristics Decrease** | | | | |
| **50% spark duration [ms]** | 38.55±0.56 (N=290) | 38.88±0.75 (N=197) | 39.66±0.69 (N=189) | 43.84±0.87** ## ^^ (N=234) |
| **Normalized amplitude [N.U]** | 0.08±0.004 (N=290) | 0.08±0.005 (N=197) | 0.08±0.004 (N=189) | 0.08±0.004 (N=234) |
| **Amplitude difference [N.U]** | 4.49±0.37 (N=285) | 3.06±0.31** (N=195) | 8.04±1.25** ## (N=187) | 5.27±0.59## ^ (N=233) |
| **Spark length [um]** | 5.47±0.11 (N=290) | 5.02±0.15* (N=197) | 4.89±0.15** (N=189) | 5.14±0.15 (N=234) |
| **LCR period [ms]** | 259.64±7.3 (N=286) | 390.95±18.34** (N=188) | 438.62±21.01** (N=175) | 637.59±27.26** ## ^^ (N=221) |
| **Number of LCR [1/sec*um]** | 29±5.22 (N=10) | 19.7±4.51* (N=10) | 18.9±3.19** (N=10) | 23.4±4.7 (N=10) |
| **Ca2+ signal of individual LCR (ms*µm*F/F0)** | 17.03±0.15 (N=290) | 16.76±0.18 (N=197) | 15.88±0.28 (N=189) | 18.49±0.27 (N=234) |
