# Supplementary material for: Ca2+-Driven Selectivity of the Effect of the Cardiotonic Steroid Marinobufagenin on Rabbit Sinoatrial Node Function
Source: Cells. 2023 Jul 18;12(14):1881. doi: 10.3390/cells12141881 (PMC10378090; doi:10.3390/cells12141881)
Supplement: Supplementary file 1 [file cells-12-01881-s001.zip › cells-2410410-supplementary/Table S3.pdf]

	Control	MBG 100nM
**Ca^{2+} transient parameters No Change**		
**Beat Interval [ms]**	512.7±46.37 (N=8)	537.28±34.29 (N=8)
**Time to peak [ms]**	129.97±15.2 (N=8)	146.37±6.79 (N=8)
**Time to 50% relaxation [ms]**	197.72±21.36 (N=8)	216.72±11.18 (N=8)
**Time to 90% relaxation [ms]**	294.76±18.88 (N=8)	338.48±23.32 (N=8)
**Spontaneous diastolic LCR Characteristics No Change**		
**50% spark duration [ms]**	39.5±0.59 (N=266)	41.15±0.6* (N=239)
**Normalized amplitude [N.U]**	2.57±0.26 (N=266)	3.39±0.37 (N=239)
**Amplitude difference [N.U]**	3.59±0.81 (N=264)	2.68±1.32 (N=236)
**Spark length [um]**	4.52±0.13 (N=266)	4.71±0.12 (N=239)
**LCR period [ms]**	297.47±8.87 (N=266)	336.83±12.52** (N=230)
**Number of LCR [1/sec*um]**	33.25±8.55 (N=8)	29.87±6.56 (N=8)
**Ca2+ signal of individual LCR (ms*µm*F/F0)**	488.23±67.34 (N=266)	721.86±100.84* (N=239)
