# Supplementary material for: Ca2+-Driven Selectivity of the Effect of the Cardiotonic Steroid Marinobufagenin on Rabbit Sinoatrial Node Function
Source: Cells. 2023 Jul 18;12(14):1881. doi: 10.3390/cells12141881 (PMC10378090; doi:10.3390/cells12141881)
Supplement: Supplementary file 1 [file cells-12-01881-s001.zip › cells-2410410-supplementary/Table S4.pdf]

	**Control**	**MBG 100nM**
**Ca^{2+} transient parameters Decrease**		
**Beat Interval [ms]**	492.18±35.47 (N=12)	744.31±103.32* (N=12)
**Time to peak [ms]**	166.3±28.4 (N=12)	150.23±23.22 (N=12)
**Time to 50% relaxation [ms]**	231.6±33.98 (N=12)	224.31±30.89 (N=12)
**Time to 90% relaxation [ms]**	324.63±34.03 (N=12)	349.78±39.16 (N=12)
**Spontaneous diastolic LCR Characteristics Decrease**		
**50% spark duration [ms]**	40.67±0.53 (N=282)	44.13±0.6** (N=305)
**Normalized amplitude [N.U]**	0.91±0.08 (N=282)	1.1±0.09 (N=305)
**Amplitude difference [N.U]**	10.75±1.39 (N=276)	6.13±0.43** (N=298)
**Spark length [um]**	4.14±0.13 (N=282)	4.07±0.09 (N=305)
**LCR period [ms]**	314.66±9.3 (N=271)	420.52±16.33** (N=291)
**Number of LCR [1/sec*um]**	23.5±3.44 (N=12)	25.41±3.55 (N=12)
**Ca2+ signal of individual LCR (ms*µm*F/F0)**	134.27±13.46 (N=282)	199.88±18.61** (N=305)
