# Supplementary material for: Ca2+-Driven Selectivity of the Effect of the Cardiotonic Steroid Marinobufagenin on Rabbit Sinoatrial Node Function
Source: Cells. 2023 Jul 18;12(14):1881. doi: 10.3390/cells12141881 (PMC10378090; doi:10.3390/cells12141881)
Supplement: Supplementary file 1 [file cells-12-01881-s001.zip › cells-2410410-supplementary/Table S5.pdf]

	Control	BAPTA 10µM	BAPTA 10µM + MBG 100nM
**Ca^{2+} transient parameters**			
**Beat Interval [ms]**	489.52±23.99 (N=12)	1257.85±147.51** (N=12)	1326.57±154.02** (N=12)
**Beat Interval SD [ms]**	53.68±7.04 (N=12)	289.05±138.45 (N=12)	186.23±43.68** (N=12)
**Time to peak [ms]**	113.53±10.15 (N=12)	284.82±24.39** (N=12)	256.54±26.92** (N=12)
**Time to 50% relaxation [ms]**	192.05±18.93 (N=12)	421.29±35.13** (N=12)	353.36±35.3** (N=12)
**Time to 90% relaxation [ms]**	313.69±30.57 (N=12)	770.38±73.81** (N=12)	708.02±57.35** (N=12)
**Spontaneous diastolic LCR Characteristics**			
**50% spark duration [ms]**	41.9±0.55 (N=349)	48.33±0.71** (N=218)	47.33±0.88** (N=167)
**Normalized amplitude [N.U]**	0.4±0.03 (N=349)	0.53±0.05** (N=218)	0.49±0.05 (N=167)
**Amplitude difference [N.U]**	4.23±0.29 (N=347)	1.24±0.12** (N=215)	2.13±0.23** ## (N=161)
**Spark length [µm]**	5.38±0.09 (N=349)	6.24±0.14** (N=218)	3.72±0.11** ## (N=167)
**LCR period [ms]**	302.64±8.96 (N=276)	777.7±28.65** (N=212)	755.17±29.74** (N=162)
**Number of LCR [1/sec*µm]**	29.08±4.86 (N=12)	18.16±2.89 (N=12)	13.91±2.73* # (N=12)
**Ca^{2+} signal of individual LCR (ms*µm*F/F0)**	94.12±8.37 (N=349)	161.4±16.33** (N=218)	80.42±9.07## (N=167)
